# Working conditions and reproductive health among healthcare professionals: Insights from a pilot study

**DOI:** 10.18332/ejm/218635

**Published:** 2026-08-07

**Authors:** Tina Gogova, Polona Ana Mivšek, Nina Jančar

**Affiliations:** 1 Faculty of Health Sciences, University of Ljubljana, Ljubljana, Slovenia; 2 Faculty of Medicine, University of Ljubljana, Ljubljana, Slovenia; 3 Division of Obstetrics and Gynecology, University Medical Centre, Ljubljana, Slovenia

**Keywords:** reproductive health, working conditions, healthcare professionals occupational exposure, preconception health

## Abstract

**INTRODUCTION:**

Despite rising awareness of occupational health, preconception health remains overlooked, particularly in demanding healthcare environments. The pilot study aimed to examine the associations between working conditions, workplace-related risks and the reproductive health of nurses and midwives.

**METHODS:**

A cross-sectional pilot study was conducted in Slovenia in 2022, including 41 participants. Associations between occupational factors and reproductive health outcomes were analyzed using descriptive statistics, the Mann-Whitney U test and the likelihood-ratio χ² test (G²), based on Kullback–Leibler divergence.

**RESULTS:**

Findings suggest possible associations between factors such as single shift work, overtime, lifting and work stress and reproductive problems. Discrimination by patients, relatives and colleagues was significantly associated with sexual desire, frequency of sexual activity and general sexual behavior. Among participants, 34.2% reported menstrual problems, 29.2% conception difficulties and 44.7% reproductive diagnoses. Post hoc effect size analysis revealed six large and two medium effect sizes, indicating potential clinical relevance beyond mere statistical significance.

**CONCLUSIONS:**

These preliminary findings indicate that certain occupational and psychosocial stressors may be linked to adverse reproductive outcomes among healthcare professionals. Addressing these risks through targeted preventive strategies could help protect the reproductive health of female healthcare professionals, most of whom are of reproductive age.

## INTRODUCTION

Preconception healthcare involves providing biomedical, behavioral, and social health interventions to women and their partners prior to conception, aiming to reduce maternal and child mortality and morbidity^1^. Although preconception health is gaining recognition, its role in occupational health research, particularly in nursing and midwifery, remains under-researched. Crucially, while much emphasis is placed on educating healthcare professionals about preconception care, little attention is given to their own preconception well-being.

The term ‘healthcare professionals’ is used throughout this article to refer specifically to nurses and midwives.

Given the large number of working women of childbearing age, the workplace has been identified as an important setting for improving women’s health and wellbeing during the preconception, pregnancy and postpartum periods^2^. Consequently, most industrialized countries have introduced maternity protection legislation to protect the health of pregnant workers and their unborn children from workplace exposure^3^ . However, there are still no comparable guidelines for maintaining the reproductive health of women of reproductive age, regardless of whether they intend to start a family.

Nevertheless, the work environment can have an impact on reproductive health, fertility, conception, pregnancy and childbirth^4^. Healthcare professionals face multiple risks, including ergonomic (prolonged standing, heavy lifting), physical (vibration, radiation), chemical (exposure to antineoplastic agents or hazardous substances), biological (viruses), organizational (shift work, irregular working hours) and psychosocial risks (work-related stress)^5-10^, to which they may be exposed simultaneously. Studies have linked these exposures to a number of reproductive health problems, including menstrual cycle disorders^11^, reduced fertility^12^ or infertility^13^, miscarriage^14^, fetal abnormalities^15^, preterm labour^14^ and stillbirth^16^, among others.

This topic is particularly important because the nursing and midwifery workforce is predominantly female, with a significant proportion of women of childbearing age. In the United States, >4.9 million nurses are employed, about half of whom are women of childbearing age^17^. Similarly, in England, almost 90% of healthcare professionals are women, and most of them are expected to have children at some point during their career^18^, but there is little data on Slovenia. While members of both professional groups play an active role in promoting reproductive health, it is important to investigate whether the demands and exposures inherent to their work may pose risks to their own reproductive health and fertility.

To begin addressing these questions, we conducted a pilot study. The primary objective of this work is to present the initial, descriptive findings regarding the associations between working conditions, workplace-related risks, and the reproductive health of nurses and midwives in Slovenia, and to discuss the methodological implications for a future, comprehensive study. Ultimately, this research aims to raise awareness and promote necessary changes and preventive measures to protect the reproductive and sexual health of healthcare professionals, because every worker deserves a safe working environment that does not compromise their ability to start a family.

## METHODS

This study was conducted and reported in accordance with the Strengthening the Reporting of Observational Studies in Epidemiology guidelines.

### Study design

A cross-sectional pilot study was conducted to explore the potential relationships between working conditions and reproductive health among healthcare professionals.

### Sampling and setting

This pilot study was conducted online without restriction to a specific healthcare institution. Participants were recruited through non-probabilistic convenience sampling combined with snowball sampling. The initial invitation was sent by email to a small group of healthcare professionals known to the research team. These individuals were asked to share the survey link within their professional and social networks.

In accordance with the predefined inclusion criteria, only those participants who completed at least 80% of the questionnaire and were actively employed in nursing or midwifery at the time of the study were included in the final analysis. Gender, possible infertility problems or treatment for fertility problems did not constitute exclusion criteria. The only exclusion criterion was not being currently employed in healthcare, despite having adequate educational background.

### Data collection and instrument development

Data was collected using an anonymous online questionnaire on the 1KA.si online platform, between June and December 2022. The questionnaire was developed by the authors of this study on the basis of a preliminary systematic literature review. A panel of experts evaluated the content and the authors edited and reviewed the final version of the survey.

On the first page of the questionnaire, participants were presented with a written informed consent form that included the purpose of the research and an emphasis on voluntary participation. By clicking the ‘Next’ button, participants expressed their consent to participate.

The questionnaire contains 62 questions, mainly closed questions with predefined answers, as well as some open sub-questions based on facts from the literature and findings of the researchers. The questionnaire consists of seven sections: 1) demographic data (gender, education level, age, etc.); 2) various dimensions of the work environment and work characteristics (shift/night work, working overtime); 3) the perceived relationship between work circumstances on workers’ lives and lifestyles; 4) assessment of working conditions and exposure to various situations at work, as well as questions on job and life satisfaction; 5) respondents’ lifestyles and sleep patterns: 6) data on reproductive health (conception, pregnancy, childbirth and children) and, for women, statements on menstruation; and 7) questions on infertility (for participants undergoing infertility diagnosis and/or treatment).

### Measurement scales, scoring and reliability

The items were rated on a four-point Likert scale. Exposure to different situations or risk factors was rated as follows: 1 ‘never’, 2 ‘occasionally’, 3 ‘very often’, and 4 ‘every day’. Higher scores mean that healthcare workers are more exposed to the listed risk factors. Compliance with safety regulations in the workplace was rated as 1 ‘not at all true’, 2 ‘not true’, 3 ‘true’ and 4 ‘very true’. The success of balancing work and personal life was rated as 1 ‘unsuccessful’, 2 ‘satisfactory’, 3 ‘good’ or 4 ‘excellent’. The assessment of the perceived relationship between work and family relationships, the quality of leisure time, the amount of physical activity, the experience of stress, fatigue, burnout, unhealthy habits and sexuality was rated as 1 ‘no influence at all’, 2 ‘no influence’, 3 ‘some influence’ and 4 ‘high influence’. In the sections related to reproductive health and infertility, we used a combination of nominal (e.g. family decision, menstrual cycles, reproductive diagnoses, type of infertility, impact on work), ordinal (time to conception), and ratio scales (e.g. age at family decision, reproductive outcomes).

The validity of the questionnaire was checked from the perspective of the substantive validity of the items, which were designed based on a literature review and expert judgment (face and content validity), and with descriptive statistics of individual statements, which indicate the diversity of the participants’ experiences and exposure. Internal consistency was assessed using Cronbach’s alpha. First, we examined the reliability of all items assessing exposure to risk factors (ergonomic loads, exposure to hazardous substances, disinfection, and compliance with safety regulations). The overall scale demonstrated acceptable reliability (α=0.73). Given the heterogeneity of the content, we subsequently analyzed the subscales. The ergonomic factors subscale (repetitive movements, bending, awkward/forced postures, lifting/moving, sudden rapid movements) showed acceptable reliability (α=0.71). The exposure to hazardous substances subscale (ionizing and non-ionizing radiation, anesthetic gases, cytostatic drugs, intravenous antibiotic administration, inhalations) also indicated acceptable reliability (α=0.72). The disinfection and compliance subscale (cleaning and disinfection of equipment and surroundings, work with highly effective disinfectants) demonstrated good reliability (α=0.81). Overall, the subscales demonstrated acceptable to good internal consistency.

### Statistical analysis

The statistical analysis of the data was performed using SPSS software version 27. The results were calculated using descriptive statistics, where frequencies, percentages, minimum and maximum values, mean and standard deviation were calculated based on the variable level. Given the small sample size and the ordinal nature of many variables, non-parametric tests such as the Mann-Whitney U test and exploratory measures such as Kullback-Leibler divergence were used to assess potential associations. A significance level of p≤0.05 was used for statistical significance.

### Post hoc effect size analysis and statistical validation

To further evaluate the robustness and practical significance of the results, additional *post hoc* analyses were performed. To assess clinical significance of associations independent of sample size, we calculated Cramér’s V effect sizes for all likelihood-ratio χ² (G²) tests, following Cohen’s conventions: V<0.1 (negligible), 0.1–0.3 (small), 0.3–0.5 (medium), and ≥0.5 (large). Given the small sample size (n=41) and violations of chi-squared assumptions due to sparse contingency tables, all significant findings were additionally validated using Fisher’s exact test. *Post hoc* power analysis determined sample sizes required to achieve 80% statistical power (α=0.05) for detecting observed effect sizes, providing empirical targets for confirmatory studies.

## RESULTS

### Basic characteristics of the healthcare professionals

The pilot study included 41 female nurses and midwives who completed at least 80% of the questionnaire, with a mean age of 36.3 years (SD=7.95; range: 23–63). The majority of respondents work as registered nurses (43.9%), followed by registered midwives (31.7%) and nursing technicians (24.4%), all of whom work in healthcare. A total of 56.1% of participants held a higher vocational or university degree, 19.5% a Master’s degree, and 2.4% a doctoral degree. A total of 19.5% of respondents are employed at the primary level, 29.3% at the secondary level, and 51.2% at the tertiary level of healthcare.

Participants’ years of employment ranged from 1 to 40, with a mean of 13.44 years (SD=8.17). Most work multiple shifts, typically 40 to 50 hours per week and spend about half of their working hours standing (Table 1).

**Table 1:** Work environment characteristics of nurses and midwives, Slovenia, 2022 (N=41)

*Characteristics*	*n*	*%*
**Shift schedule**		
Day work	8	19.5
Rotating and night shifts	33	80.5
**Working hours per week**		
20	1	2.4
30	1	2.4
40	13	31.7
41–50	22	53.7
>50	4	9.8
**Working position**		
Sitting	5	12.2
Standing	12	29.3
I spend about half of my working time standing	24	58.5

Table 2 shows the level of workplace exposure to various factors, rated from 1 (never exposed) to 4 (exposed daily). Descriptive analysis indicates that, on average, respondents are frequently exposed to tasks such as cleaning and disinfecting equipment, using high-level disinfectants in patient areas, decontaminating instruments and performing repetitive movements, bending and awkward postures. They are regularly involved in lifting or moving patients, handling heavy loads and preparing and administering intravenous antibiotics. Occasionally they are exposed to sudden movements, ionizing radiation, preparing inhalations, yet exposure to cytostatic drugs, anesthetic gases, and non-ionizing radiation is rare.

**Table 2:** Level of occupational exposure of healthcare professionals, Slovenia, 2022 (N=41)

*Exposures at work*	*Min*	*Max*	Mean*	*SD*
Cleaning and disinfecting equipment and devices in patient areas	1	4	3.44	0.84
Working with highly effective disinfectants	1	4	3.29	0.98
Performing repetitive movements	1	4	3.02	0.88
Bending	1	4	2.95	0.84
Unfavorable or coercive posture	2	4	2.93	0.85
Decontamination and disinfection of instruments or other devices	1	4	2.73	0.98
Transferring/lifting patients (without modern equipment)	1	4	2.54	0.93
Preparing and administering antibiotics intravenously	1	4	2.46	1.16
Sudden movements (e.g. patient fall, resuscitation)	1	4	2.29	0.72
Preparing and administering inhalations	1	4	2.02	1.19
Ionizing radiation (X-ray, CT, nuclear medicine, radiotherapy)	1	4	1.68	0.88
Transferring/lifting patients using modern equipment	1	3	1.54	0.71
Cytostatic drugs	1	4	1.39	0.83
Administering cytostatic drugs	1	4	1.29	0.84
Anesthetic gases	1	3	1.27	0.55
Non-ionizing radiation (MR)	1	3	1.22	0.48
Preparation of cytostatic drugs	1	3	1.1	0.37

CT: computed tomography. MR: magnetic resonance.

* Scale: 1=never, 2=occasionally, 3=very often, 4=every day.

### Reproductive health of healthcare professionals according to working conditions

The study found that most participants were unaware of the risks to reproductive health in the workplace; 97.6% stated that they had not received counseling on this topic from their employer. Participants mentioned several workplace factors that they considered to be associated with reproductive health risks, including exposure to radiation, stress, physical strain, heavy lifting, night and shift work, handling cytostatic drugs and hazardous drugs, and mentally demanding work. A statistically significant association was identified between the participants’ self-reported awareness of workplace hazards and preventive behavior and the occurrence of conception problems. Individuals who reported a higher level of awareness and preventive behavior had a lower prevalence of conception problems (χ^2^=6.932; df=2; p=0.031).

Respondents were asked to indicate any reproductive diagnoses they had been diagnosed with (blocked fallopian tubes, PCOS, endometriosis, abnormal PAP results, HPV infection, hormonal imbalances) as well as menstrual or fertility problems. The results of the study suggest that the occurrence of reproductive problems may be associated with various workplace-related factors. We observed a trend towards a statistically significant association between single-shift work and the diagnosis of reproductive problems (χ^2^=3.831; df=1; p=0.050), while working hours, particularly among participants who worked 40 to 50 hours per week, were statistically significantly associated with menstrual problems (χ^2^=14.248; df=4; p=0.007). Furthermore, a statistically significant association was found between menstrual problems and the lack of use of modern equipment for moving or lifting patients (χ^2^=8.001; df=2; p=0.018). However, it should be noted that the results are limited in terms of reliability and validity due to the small sample size and the disproportionate representation of the groups.

The biggest psychological stressors for participants in the workplace were demanding patients or their relatives (85.4%), work-related stress (75.6%), challenging medical conditions of the patient (68.3%), staff shortages (65.9%), and decision-making demands (61%). Approximately half felt stressed by relationships between staff (51.2%), while fewer cited difficult working conditions (43.9%), dealing with the death of patients (41.5%), and inappropriate working conditions (39%) as burdens. The pilot study revealed a statistically significant association between experiencing stress and the presence of problems with conception, with conception difficulties more commonly reported by women who experienced work-related stress (χ^2^=4.918; df=1; p=0.027).

### Exposure to violence or discrimination in the workplace

Most participants stated that they had been exposed to verbal violence at work (58.5%), discrimination (29.3%), and psychological harassment by patients or their relatives (26.8%). Discrimination in the workplace by colleagues is the most commonly reported issue (19.5%), followed by mobbing (17.1%), verbal violence and psychological harassment (both 9.8%). Physical violence and sexual harassment by colleagues were not reported, while sexual harassment of employees by patients or their relatives was reported in 4.9%, and physical violence in 14.6%. In our study, discrimination by patients or their relatives was significantly associated with the perceived influence of work on participants’ sexual desire, frequency of sexual intercourse and changes in sexual habits. Employees who reported discrimination were more likely to indicate that their work influenced these aspects of their sexuality compared to those who did not experience discrimination (Table 3). However, the direction of this influence (i.e. whether positive or negative) was not assessed in our study.

**Table 3:** Association between discrimination by patients or relatives and employees’ sexuality, Slovenia, 2022 (N=36)

*Employees‘ sexuality*	*Discrimination by patients or relatives*	Level of employees’ sexuality*							
		*1*	*2*	*3*	*4*	*Total*			
		*n (%)*	*n (%)*	*n (%)*	*n (%)*	*n*	*χ^2^*	*df*	*p*
**Frequency of sexual intercourse**	No	8 (33.3)	5 (20.8)	10 (41.7)	1 (4.2)	24	11.358	3	0.010
	Yes	0 (0.0)	1 (8.3)	11 (91.7)	0 (0.0)	12			
**Total**		8 (22.2)	6 (16.7)	21 (58.3)	1 (2.8)	36			
**Change in sexual habits**	No	8 (33.3)	9 (37.5)	6 (25.0)	1 (4.2)	12	10.659	3	0.014
	Yes	0 (0.0)	4 (33.3)	8 (66.7)	0 (0.0)	24			
**Total**		8 (22.2)	13 (36.1)	14 (38.9)	1 (2.8)	36			
**Sexual desire**	No	10 (41.7)	6 (25.0)	8 (33.3)		24	12.101	2	0.002
	Yes	0 (0.0)	2 (16.7)	10 (83.3)		12			
**Total**		10 (27.8)	8 (22.2)	18 (50.0)		36			

* Level: 1=no influence at all, 2=no influence, 3=some influence, 4=high influence.

Psychological harassment by colleagues was significantly associated with sexual desire and change in sexual habits (χ^2^=6.046; df=2; p=0.049), while discrimination by colleagues was associated with feelings of burnout (χ^2^=14.826; df=3; p=0.02).

The strongest associations between working conditions and participants’ lives were observed on fatigue, the ability to fulfil family obligations and the quality of leisure time (Table 4).

**Table 4:** Associations between work conditions and participants’ lives, Slovenia, 2022 (N=36)

*Conditions*	*Min*	*Max*	Mean*	*SD*
Fatigue	2	4	3.19	0.58
Family obligations	2	4	3.03	0.56
Quality leisure time	2	4	2.89	0.62
The amount of physical activity	2	4	2.86	0.54
Perceived work-related stress	1	4	2.86	0.72
Burnout	1	4	2.81	0.95
The quality of participants’ family or partner relationships	1	4	2.75	0.81
The frequency of sexual intercourse	1	4	2.42	0.87
Sexual desire	1	3	2.22	0.87
Changes in sexual habits	1	4	2.22	0.83
Drinking alcohol	1	4	1.42	0.73
Smoking	1	4	1.28	0.70
The use of prohibited substances	1	4	1.19	0.62

* Scale: 1=never, 2=occasionally, 3=very often, 4=every day.

To address the research question of whether the length of employment and prolonged exposure to risk factors were associated with an increased likelihood of reproductive problems, those factors were examined but found no significant differences. The results of the Mann-Whitney U-test showed no association with reproductive diagnosis (U=159.50, p=0.577), duration of conception (U=48.00, p=0.464), or menstrual problems (U=161.00, p=0.963).

### Effect sizes and statistical validation

Table 5 shows that *post hoc* effect size analysis revealed that several associations between occupational factors and reproductive health outcomes demonstrated medium to large effects, suggesting potential clinical importance beyond statistical significance. Six associations achieved large effect sizes (Cramér’s V ≥0.5) and two associations showed medium effect sizes (0.30 ≤ V < 0.50). This additional analysis indicates that observed associations are robust and are not artifacts of small sample statistical issues (Supplementary file Figure 1).

**Table 5:** Significant associations between occupational factors and reproductive health outcomes, Slovenia, 2022

*Associations*	*N*	*χ^2^*	*df*	*p*	*Cramer’s V*	*Effect size*	Required sample*
Work stress → conception difficulties	24	4.918	1	0.027	0.45	Medium	16
Discrimination (colleagues) → burnout	36	14.826	3	0.02	0.64	Large	19
Preventive behavior → conception success	24	6.932	2	0.031	0.54	Large	30
Discrimination (patients) → sexual desire	36	12.101	2	0.002	0.58	Large	31
Discrimination (patients) → sexual frequency	36	11.358	3	0.01	0.56	Large	33
Discrimination (patients) → sexual habits	36	10.659	3	0.014	0.54	Large	35
Modern equipment → menstrual problems	38	8.001	2	0.018	0.46	Medium	43
Overtime work (>40 h) → menstrual problems	38	14.248	4	0.007	0.61	Large	47

* Required sample size to achieve 80% statistical power (α=0.05) based on observed effect size. All significant associations were cross validated using Fisher’s exact test due to chi-squared assumption violations.

## DISCUSSION

The preliminary results of the pilot study suggest a possible association between the working environment and reproductive problems, indicating the need for a larger study to confirm these findings and to provide data on the prevalence of infertility among healthcare professionals in Slovenia.

A supportive and conducive workplace environment enhances staff well-being and improves patient care quality, safety and satisfaction^19^. In our opinion, equally important are employees’ personal values and their awareness of responsible behavior. A literature review found that fertility awareness among people of reproductive age is generally low to moderate^20^. This is consistent with our findings, as most participants were unaware of workplace reproductive risks and reported not receiving employer-provided training. This low awareness was unexpected, as participants’ high level of education might have been assumed to independently ensure greater knowledge and awareness of such risks, regardless of the lack of employer-provided training.

Our findings, although preliminary, suggest that awareness is an important protective factor. Following established guidelines, helps optimize patient care by improving safety, quality, and consistency in healthcare^21^. It can be assumed that, to encourage protective behaviors, employees must first be aware of potential hazards, especially those related to reproduction, which are often overlooked. This is supported by our results, which suggest that employees who are aware of workplace risks and take precautions, tend to have fewer reproductive problems.

This is confirmed also by a study that examined the administration of antineoplastic drugs to nurses planning a pregnancy. No association with fertility impairment was found, especially in those who consistently applied exposure control measures (use of gloves, gowns and needle-free systems) or took steps to reduce or eliminate exposure to hazardous substances^14^. Another study found that women who were frequently exposed to high-level disinfectants had less fertility impairment, probably because 90% of women used protective equipment compared to 62% of women with lower exposure. Those who never used protective equipment had more than double duration of pregnancy attempts^12^. This confirms the importance of healthcare professionals being informed about their potential occupational risks.

However, the observed associations between work-related risk factors and reproductive health outcomes may be explained by several organizational, ergonomic and biological mechanisms. The healthcare sector is characterized by irregular schedules, including night and rotating shifts, long hours, prolonged standing and heavy lifting^8,22^. Prolonged standing, frequent heavy lifting, night work, anti-cancer drug exposure, and overtime work were found to be moderately associated with menstrual disorders^23^. In another study in which 41% of nurses experienced menstrual disorders, they identified that handling disinfectants was the most significant risk factor, followed by high workload, and the nursing profession itself^10^.

Strenuous work schedules may also be associated with fertility disorders, though current evidence remains limited and inconclusive^8^. One study reported that long working hours (>40 hours/week) and heavy lifting (>15 times/day) increased the time to conception by 20% and by 49% among overweight women. In contrast, night shifts had no significant effect^7^.

Several occupational exposures have been associated with adverse pregnancy outcomes. Prolonged standing and heavy workloads were linked to preterm delivery and low birth weight^22^. A systematic review confirmed that lifting ≥10 kg more than ten times per day was associated with an increased risk of spontaneous abortion and preterm delivery^24^. Chemical and ergonomic exposures were associated with stillbirth and spontaneous abortion^25^. In contrast, some studies on shift work and fertility outcomes have reported no significant differences^25,26^. While the effects of occupational exposure on pregnancy have been extensively researched, its association with preconception health remains underexplored, requiring further research.

In Europe, women constitute 75% of the healthcare workforce^27^. As such, preconception care should be provided to all women of childbearing age, regardless of their pregnancy intentions^28^, especially since pregnancy is typically not recognized until several weeks after conception.

Working overtime can lead to various consequences affecting pregnancy, including less time to sleep and recover from work, increased exposure to occupational hazards and demands, and diminished time for family, leisure and exercise^29^. Similar associations were observed in our study. Participants reported that work had the greatest association with fatigue, family responsibilities, leisure time, physical activity, stress, and burnout. It also significantly affected their relationships with their partners. Moreover, workplace stress extends beyond professional responsibilities, influencing personal life and affecting relationships^30^. Therefore, a strong and supportive partnership is essential to help balance work and personal life and mitigate the negative effects of work-related stress on wellbeing and reproductive health. In addition, factors such as long working hours, shift work, lack of social support and poor work–life balance, all common contributors to burnout, have been independently associated with adverse reproductive outcomes, including infertility and adverse pregnancy outcome^31,32^ Nursing consistently ranks among the most stressful healthcare professions, placing healthcare professionals at particularly high risk for occupational burnout^33^. Moreover, providing a healthier work environment not only supports staff well-being but also leads to greater job satisfaction, improved performance, higher quality patient care, and better organizational outcomes^34^.

Our analysis also identified a statistically significant association between discrimination by patients or their relatives and changes in employees’ sexual desire, frequency of sexual intercourse, and sexual habits. Workplace stress, conflict, and exposure to violence may also negatively affect family and partner relationships, including aspects of sexual health. This highlights the significant emotional and psychological toll of workplace discrimination, affecting well-being, relationships, and sexuality, which could also influence conception. These findings underline the need for improved support systems in the workplace.

Professional relationships, both between employees and with patients and their families, are becoming increasingly challenging. Stress in the work environment is not only triggered by demanding interactions with patients and their families, but also by interpersonal conflicts with colleagues. The study found that nurses who faced high levels of stress due to conflicts with colleagues and patients, are more likely to experience work–family conflict. This suggests that workplace stress, including conflicts or violence, can spill over into personal life, exacerbating stress and conflict outside of work^35^. This is concerning, as our results revealed a statistically significant association between work-related stress and difficulties with conception, especially among women experiencing workplace stress. A survey conducted among healthcare workers in Slovenia revealed that 56.5% of respondents experienced high levels of stress^36^.

Interestingly, despite these risks, no association was found between the length of employment and reproductive problems. This suggests that the type and intensity of occupational exposures, rather than duration alone, could be more critical and needs further investigation.

To protect the reproductive health of healthcare professionals, it is crucial to increase their awareness and provide education on occupational risk factors. Employers should support this by implementing effective protective measures in the workplace.

### Limitations

This study has some limitations. First, its cross-sectional design precludes conclusions about causality and raises the possibility of reverse causality. Second, as a self-report-based study, response bias may have affected results, including recall bias for past behaviors and social desirability bias for positive or negative behaviors. Third, as a pilot study, selection bias cannot be ruled out, participants concerned about reproductive health may have been more motivated to participate, reducing sample representativeness. Fourth, the small sample limited statistical power, subgroup analyses and generalizability of the results. Given sparse contingency tables, effect sizes were emphasized over p-values, and significant associations were validated with Fisher’s exact test to ensure robustness. Given these methodological limitations, the results should be interpreted with caution and considered primarily as preliminary findings that provide a basis for future studies with larger, more representative samples.

### Future research

As this was a pilot study, the focus was on descriptive associations rather than causal modelling. Future studies with larger, representative samples should employ more advanced statistical analyses accounting for sociodemographic and occupational factors to provide more precise estimates. Larger studies are needed to develop recommendations for improving working conditions affecting fertility and raise awareness among healthcare professionals about the potential associations between occupational exposures and their reproductive health and sexual well-being.

## CONCLUSIONS

Ensuring a safe working environment is essential to support the reproductive health and fertility of healthcare workers, given the observed associations between occupational exposures and reproductive outcomes. Although the sample size was limited, the medium to large effect sizes and the results of Fisher’s exact test indicate clinically relevant associations that warrant confirmation in larger studies. These findings highlight the importance of developing workplace policies and preventive measures that mitigate occupational risks and promote the well-being of female healthcare professionals.

## Supplementary Material



## Data Availability

The data supporting this research are available from the authors on reasonable request.
