# Supplementary material for: Working conditions and reproductive health among healthcare professionals: Insights from a pilot study
Source: Eur J Midwifery. 2026 Aug 7;10:10.18332/ejm/218635. doi: 10.18332/ejm/218635 (PMC13492245; doi:10.18332/ejm/218635)
Supplement: Supplementary file 1 [file EJM-10-34-s1.pdf]

Supplementary Figure 1: Effect sizes for occupational factors and reproductive health

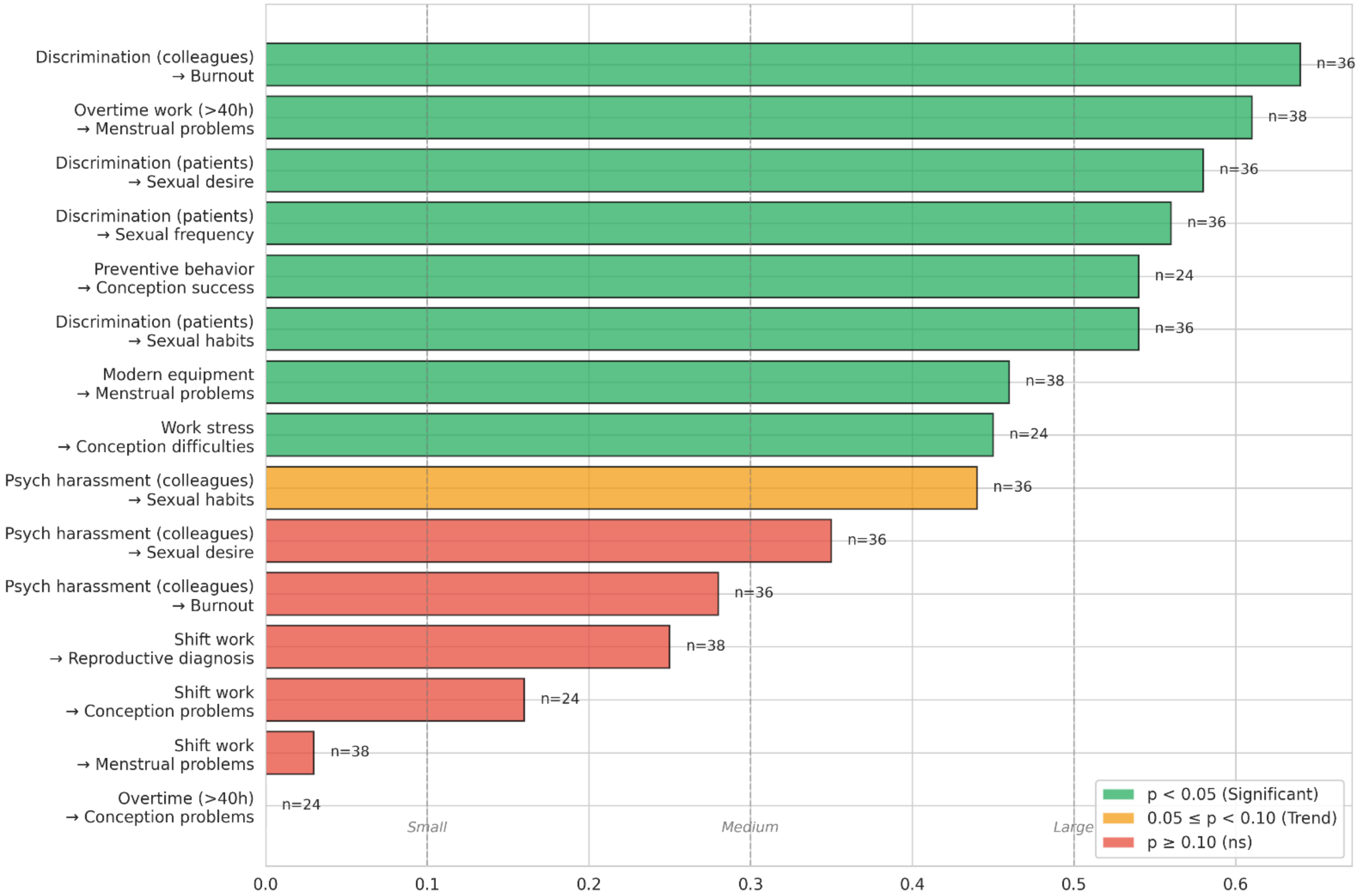

Effect sizes (Cramér's V) for occupational factor associations with reproductive health outcomes. Bars represent effect size magnitude. Colors indicate statistical significance validated by Fisher's exact test: green ($p<0.05$), orange ($0.05 \leq p<0.10$), red ($p \geq 0.10$). All significant associations (green) were confirmed by Fisher's exact test despite chi-square assumption violations.

© 2026 Gogova T. et al.
